# Therapeutic Development of Mesenchymal Stem Cells or Their Extracellular Vesicles to Inhibit Autoimmune-Mediated Inflammatory Processes in Systemic Lupus Erythematosus

**DOI:** 10.3389/fimmu.2017.00526

**Published:** 2017-05-10

**Authors:** Juhi Sharma, Jeffrey M. Hampton, Giancarlo R. Valiente, Takuma Wada, Holly Steigelman, Matthew C. Young, Rachel R. Spurbeck, Alisa D. Blazek, Steffi Bösh, Wael N. Jarjour, Nicholas A. Young

**Affiliations:** ^1^Division of Rheumatology and Immunology, Department of Internal Medicine, Wexner Medical Center at The Ohio State University, Columbus, OH, USA; ^2^The Inflammation Foundation, Orlando, FL, USA; ^3^Battelle Memorial Institute, Columbus, OH, USA; ^4^Université de Nantes, Immuno-endocrinologie Cellulaire et Moléculaire, Nantes, France

**Keywords:** systemic lupus erythematosus, extracellular vesicles, mesenchymal stem cells, autoimmunity, inflammation, emerging therapies

## Abstract

Since being discovered over half a century ago, mesenchymal stem cells (MSCs) have been investigated extensively to characterize their cellular and physiological influences. MSCs have been shown to possess immunosuppressive capacity through inhibiting lymphocyte activation/proliferation and proinflammatory cytokine secretion while simultaneously demonstrating limited allogenic reactivity, which subsequently led to the evaluation of therapeutic feasibility to treat inflammatory diseases. Although regulatory constraints have restricted MSC development pharmacologically, limited clinical studies have shown encouraging results using MSC infusions to treat systemic lupus erythematosus (SLE); but, more trials will have to be performed to conclusively determine the clinical efficacy of MSCs to treat SLE. Moreover, there are some data to suggest that MSCs possess tumorigenic potential and that the immunosuppressive influence can be dramatically affected by both donor variability and *ex vivo* expansion. Given that recent studies have found that the immunosuppressive effects of MSCs are a result, at least in part, to extracellular vesicle (EV) secretion, the use of MSC-derived EVs has been suggested as a cell-free therapeutic alternative. Despite the positive data observed using EVs isolated from human MSCs to suppress inflammatory responses *in vitro* and in inhibiting autoimmune disease pathogenesis in preclinical work, there are no studies to date examining EVs from MSCs to treat SLE in humans or animal models. Considering that EVs are not subject to the strict regulatory constraints of stem cell-based pharmacological development and are more readily standardized with regard to industrial-scale production and storage, this review outlines the anti-inflammatory biology of MSCs and the scientific evidence supporting the potential use of EVs derived from human MSCs to treat patients with SLE.

## Introduction

Mesenchymal stem cells (MSCs) are stem cells that retain multipotency and have robust immunoregulatory influence. MSCs can affect many different immune cell subtypes of both the innate and adaptive immune systems. Previous studies have demonstrated that MSCs can suppress proliferation of T-cells, B-cells, natural killer cells, and dendritic cells (DCs) in a dose-dependent manner (1–3). Furthermore, MSCs have the ability to direct macrophages to a more immunotolerant phenotype that is characterized by alternative activation (4). Cytokine secretion profiles of T and B-cells are also significantly altered to a less inflammatory phenotype by incubation with MSCs, which can further contribute to the observed immunosuppressive properties (5). In addition to suppression of proinflammatory cells, MSCs can stimulate the production of regulatory T-cells (Tregs) that can inhibit inflammatory responses (6). Collectively, these data characterize MSCs as active suppressors of inflammation that effectively modulate the immune response at many levels.

Since MSCs are mainly found in the bone marrow (BM), a distinct physiological association with the immune system is evidenced despite not having hematopoietic origin (7). In addition to BM, MSCs can also be isolated from multiple fetal and adult tissue sources, including placenta, umbilical cord (UC), amniotic fluid, Wharton’s jelly, adipose tissue, and dental tissues (8, 9), which makes MSCs one of the most accessible primary cell subtypes and an attractive candidate to develop therapeutically.

Systemic lupus erythematosus (SLE) is a prototypic and chronic autoimmune disease resulting in a multi-organ inflammatory response that can cause significant morbidity and mortality without medical intervention using immunosuppressive agents (10). The autoimmune-mediated inflammatory responses observed in SLE are characterized by autoantibody complex formation and proinflammatory cytokine induction that result in activation of cells of both the innate and adaptive immune systems. The pathogenesis of SLE is complex and most likely influenced by a combination of genetics, environment, and hormonal factors. In our research, we have demonstrated that estrogen can lower the threshold of immune cell activation and induce the upregulation of a unique set of genes that may be involved in autoimmune-mediated pathology, including toll-like receptor (TLR)8, STAT1, and ZAS3 (11, 12). Despite considerable time and research over many years in our laboratory and others, the mechanism of SLE pathogenesis still remains largely elusive. Consequently, only one SLE-targeted drug has been approved by the USA Food and Drug Administration (FDA) to treat patients in more than 50 years, but the efficacy is still under extensive debate (13). Therefore, SLE patients could significantly benefit from the development of new therapies that target the autoimmune-mediated inflammatory pathology associated with this disease.

## Preclinical Studies Using MSCs to Treat SLE

To investigate the potential of MSCs to suppress autoimmune-mediated inflammatory processes, animal models were initially evaluated, and compelling experimental evidence was produced in the MRL/lpr and NZB/W F1 mouse models of SLE. To compare against conventional cyclophosphamide (CTX) treatment, MRL/lpr mice-given human BM MSC transplants showed a significant reduction in levels of anti-dsDNA and anti-nuclear autoantibodies, immunoglobulin (Ig)G, IgM, and serum albumin. Additionally, MSC therapy prevented damage to glomerular morphology/structure and reduced renal complex deposition of both complement component 3 (C3) and IgG (14). Further research investigations into this mechanism in MRL/lpr mice have shown that MSCs can suppress B-cell activation (15). Moreover, infusion of human MSCs derived from BM suppressed glomerulonephritis, decreased autoantibody production, reduced proteinuria, and improved overall survival in NZM/W F1 mice via inhibition of follicular helper cell (Tfh) development and activation of humoral immunity (16). Collectively, the data from these animal studies provided the preclinical validation necessary to move forward with human clinical trials using MSCs from healthy donors to treat SLE.

## Clinical Trials Using MSCs in Refractory SLE Patients

Over 300 refractory SLE patients have been treated at Nanjing Drum Tower Hospital in China with MSC therapy. This hospital was part of a multicenter study using two intravenous infusions of UC MSCs in patients with disease refractory to treatment and displaying active lupus nephritis (17). Their results demonstrated a well-tolerated safety profile with 32.5% (13/40) of patients achieving a major clinical response and a significant decrease in disease-activity scores. In addition, a 4-year review of both UC MSC and BM MSC transplantation in active and treatment-resistant SLE patients was published by this group to examine long-term safety and efficacy of treatment (18). Following one intravenous infusion of allogenic MSCs, there were no adverse events related to transplantation, but disease activity scores, serum autoantibody levels, and complement component fragments all significantly declined. The results from these trials indicate the efficacy of MSCs to be used as a therapeutic strategy to treat refractory SLE. Currently, there is a multicenter clinical trial (NCT02633163) in the United States that intends to establish the safety and efficacy of using UC-derived MSCs to treat SLE. This placebo-controlled, randomized, and double-blinded trial has been approved, but is not currently enrolling patients.

## MSC Secretome Can Contribute to Immunosupressive Effects

To examine if MSCs are perpetuating immunosuppressive signaling via secreted paracrine mediators rather than through cell-to-cell contact, BM-derived macrophages were stimulated with TLR7 and TLR8 ligand and cultured with either MSCs or conditioned media derived from MSCs; under both conditions and to similar extents, IL-6, TNF-α, and NFκB were significantly inhibited, and IL-10 was increased (19). The results of this study suggested that the extracellular mediators produced from MSCs may be responsible, at least in part, for the immunosuppressive effects.

Previous research has indicated that the components of the MSC secretome that may be mediating this immunosuppressive function are extracellular vesicles (EVs) (20). As defined by the International Society of Extracellular Vesicles (ISEV), EVs are spherical membrane-enclosed bodies secreted by cells and can be divided into subpopulations based on size and cellular biogenesis: exosomes (50–150 nm) originating from multivesicular bodies; microvesicles (150–1,000 nm) originating from the plasma membrane; and apoptotic bodies (>1 µm) originating from the plasma membrane of dying cells (21). However, since the sizes of the vesicles overlap, well-defined markers still lack consensus, and the precise origination of the vesicles obtained is often unknown, ISEV has suggested to use the term EV to describe the vesicles present in experimentally purified samples (22). Although identified over 30 years ago and described as a mechanism by which cells eliminate unwanted proteins/molecules (23), incontrovertible evidence generated over the past decade has now characterized the role of EVs in cell-to-cell communication (24). In support of the critical role of MSC-derived EVs in regulating *in vivo* biological activity, size fractionation analysis of conditioned media from human MSCs shown to reduce myocardial infarct sizes in pig and mouse models revealed a 50–200 nm exosomal complex originating from multivesicular intracellular endosomes as the principal bioactive component (20). Since extracellular particles of this size range can be consistently isolated from human sources and used successfully in the clinic following standardized differential ultracentrifugation protocols (25), these EVs have been the focus of recent molecular characterization (26) and therapeutic development (27).

EVs facilitate cellular communication by delivering bioactive cargo containing, among other molecules, microRNAs (miRs) that can elicit functional consequences in recipient cells (28). Results from our previous studies have demonstrated that extracellular miRs are contained primarily within EVs and that specific miRs can act as an endogenous ligand for TLR7 and TLR8, which are upregulated in SLE patients and induced by estrogen (29, 30). This novel signaling mechanism in addition to the conventional role in suppressing gene expression by targeting specific mRNA sequences make EV-encapsulated miRs potent immunoregulatory elements.

## Barriers to the Use of MSCs in Clinical Trials

Despite the positive data from human clinical trials using MSCs to treat SLE, there are no trials in the United States that are actively enrolling patients. Clinical trials will require that the MSCs are produced in an FDA-approved clean facility and that each trial is approved individually, which presents logistical and/or cost-limiting hurdles to commercial production and to the establishment of these trials. Additionally, there is some experimental evidence to suggest that MSCs have the potential to develop into sarcomas (soft tissue cancers derived from cells of mesenchymal origin) in mouse, rat, and rabbit models after transfer of MSCs cultured as early as the third passage (31–33). Moreover, *in vitro* data have demonstrated that human MCSs have oncogenic transformation potential and differentially expressed transcripts (34). In contrast, multiple studies have demonstrated that human MSCs are resistant to spontaneous transformation and clinical trials with MSCs have not revealed associations with cancer (35). Furthermore, while MSCs may promote tumorigenesis as a co-culture or in a tumor microenvironment *in vivo* at higher concentrations, a well-documented anti-tumorigenic influence has been characterized when present at a lower ratio to cancer cells (35). Also, in some studies where human MSCs have been associated with oncogenic potential, DNA fingerprinting has shown cross-contamination containing a significant percentage of misidentified cells (36). Thus, while the evidence to suggest an association of MSCs with cancer in humans is weak, the uncertainty may negatively impact MSC approval for clinical trials.

In addition to regulatory constraints and malignant potential, there are other drawbacks to using MSC therapy in SLE patients. While high numbers of MSCs can suppress lymphocyte proliferation and Th17 proinflammatory activation, lower numbers can actually elicit cellular responses to the contrary (37, 38). Treatment methods also require multiple MSC donor sources, which introduces heterogeneity to the therapeutic process. In examining BM MSC markers, significant differences were found in subpopulations of cells corresponding to gender and age of donors (39). Consequently, the inevitable donor variability leads to cell products that may greatly differ from sample to sample. In addition, a recent study has shown that population heterogeneity is also observed following long-term *in vitro* culturing of MSCs (40). Furthermore, culturing is not standardized currently for therapeutic application, and the well-characterized *ex vivo* expansion methods can cause culture-induced senescence or result in genetic drift that yields cells significantly different biologically when compared to their *in vivo* predecessors (41). These changes may ultimately lead to the loss of functional properties or produce cells with diminished immunosuppressive capacity.

In the commercial development of MSCs for clinical use, time and storage are also important considerations. Cryopreservation will be required to increase the shelf-life and provide more standardized use of MSCs, but this research is still in its infancy (42). In order for MSCs to be developed as an off-the shelf pharmacological alternative, more effective preservation will be essential to ensure that the cells can be stored long enough for safety testing, quality control evaluation, transportation to the site of administration, and coordination with the patient care regimens (43). Currently, there are no optimized methods or standardized protocols used in the development of MSC-based therapies with regard to culture and cryopreservation. Until these storage issues can be circumvented, MSCs must be isolated and processed individually for each therapeutic application, which can delay treatment. Additionally, SLE has a relatively effective standard of care and more favorable patient prognosis when compared to other diseases where MSCs are being considered therapeutically (44). Collectively, these factors may largely attribute to the absence of clinical trials using MSCs to treat SLE in the USA despite the positive data obtained from trials outside of the USA with well-tolerated safety profiles and no adverse consequences.

## Therapeutic Potential of MSC-Derived EVs to Treat SLE

Through selective protein and RNA packaging via tightly regulated cellular processes, EVs are secreted to exert functional effects in recipient cells (45). To this accord, previous studies have demonstrated potent immunosuppressive capacity with EVs derived from MSCs; specifically, EVs isolated from the conditioned media of MSCs inhibited T-cell proliferation while increasing both Treg and IL-10 levels (46). These EVs also suppressed B-cell proliferation and antibody secretion in a dose-dependent manner (47). Since human MSCs are one of the most prolific producers of EVs when compared to other cell types (48), they are conducive to industrialized production and isolation of EVs for therapy.

While the field of EV therapy to treat inflammatory disease is still emerging, some promising studies indicate therapeutic potential. EVs isolated from human MSCs have been shown to be effective in reducing kidney inflammation and maintaining joint integrity, which are both target areas of SLE pathology. In a mouse model of acute kidney injury, EV therapy prevented chronic tubular inflammation, histopathology, and damage to renal function, as determined by blood urea nitrogen and creatinine levels (49). In the first work establishing the efficacy of EVs in cartilage repair, weekly intra-articular treatment of rats with human MSC EVs in an osteochondral defect model resulted in significant improvement histologically and complete renewal of cartilage and subchondral bone (50).

In addition to preclinical studies, MSC-derived EVs have also been used successfully in the clinic to treat inflammatory disease. In this case, a patient with treatment-refractory graft-versus-host disease (GvHD) was given EV therapy from MSCs derived from a single healthy donor. Increasing doses were provided every 2–3 days without apparent side effects and analysis of patient cells incubated *in vitro* with the EVs demonstrated a 50% reduction of IL-1β, TNF-α, and IFN-α. The clinical symptoms of GvHD improved significantly within 2 weeks and were stable out to 4 months following treatment, which allowed steroid medications to be reduced significantly (51). However, despite the efficacy and clinical potential indicated by these studies, there are currently no investigations published using EVs from MSCs in SLE animal models or in human patients.

## Pharmacological Development of EVs from MSCs Offers Selective Advantages

The use of MSC-derived EVs as a cell-free therapeutic alternative offers several distinct advantages to the parent stem cells with regard to both regulatory approval and therapeutic development. EVs are not subject to FDA rules regulating the administration of living cells and have not been shown hitherto to possess carcinogenic potential themselves. In addition, EVs are highly stable and easily stored for long-term usage by simple freezing, and research from this group has shown that the addition of trehalose, a common pharmacological preservative, can enhance stability even further and permit multiple free/thaw cycles (52). EVs also have no risk of aneuploidy or other chromosomal abnormality and there are no data to indicate that EVs can have opposing immunomodulatory influences based on the amount given. Even though MSCs are well tolerated, they have been shown to elicit a detectable humoral and cellular immune response *in vivo* (53). EVs are also able to cross the blood–brain barrier while MSCs cannot, which would be particularly advantageous in the treatment of patients with central nervous system involvement leading to neuropsychiatric SLE, which is currently challenging to treat and can afflict more than half of SLE patients to some extent (54). Also, while the use of genetically manipulated cells would introduce additional regulatory review, the application of EVs produced by these cells is not subject to this regulation. According to ISEV, all EV-based therapies would be considered in accordance with the guidelines regulating medicinal products under the pharmaceutical class of biologicals (55, 56). To further facilitate standardization, MSCs have been successfully immortalized recently with little effect on the EVs and RNA/protein content packaged within (57), which could translate to industrialized EV therapy in the near future. However, this would require additional testing and validation to establish the safety of EVs produced by such cell lines.

Although the standardization of EV isolation is still lacking in the field for either therapeutic application or basic research (21), studies have shown viable strategies for development of EV-based therapy in a clinical setting. While the conventional method used for EV isolation is differential centrifugation and is historically associated with high purity compared to other techniques, the force involved in this process produces EV products with diminished functional/therapeutic capacity (58). Alternatively, chromatographic purification using size-exclusion, ion exchange, and flow field-flow fractionation techniques have been developed that yield similar purity while retaining functionality (59, 60).

## Conclusion

Using MSCs, researchers have demonstrated the feasibility of this therapeutic strategy to treat SLE in humans, with clinically measurable improvements observed in a significant number of patients. However, these clinical trials were non-randomized and in an ethnically homogenous cohort; therefore, the applicability of these findings in a more diverse patient population in a randomized clinical trial is not apparent and larger multicenter, double-blind trials will have to be examined before the effectiveness of MSCs is definitive.

The progress of using MSCs in the USA to treat SLE has lagged considerably behind due to the formidable challenges associated with drug development and safety concerns. When MSCs have been granted approval for clinical trials in the USA for other diseases, the products were from a single donor only and subject to extensive, time-consuming, and costly characterization. Since recent work indicates that the immunomodulatory effects observed by MSCs can be elicited, at least in part, by secreted paracrine factors, EVs have been explored as the mediator of this response and have shown promising results. However, as with all new therapeutic approaches, there are unknowns that will need to be examined and methodologies to be characterized and standardized.

**Figure 1:**
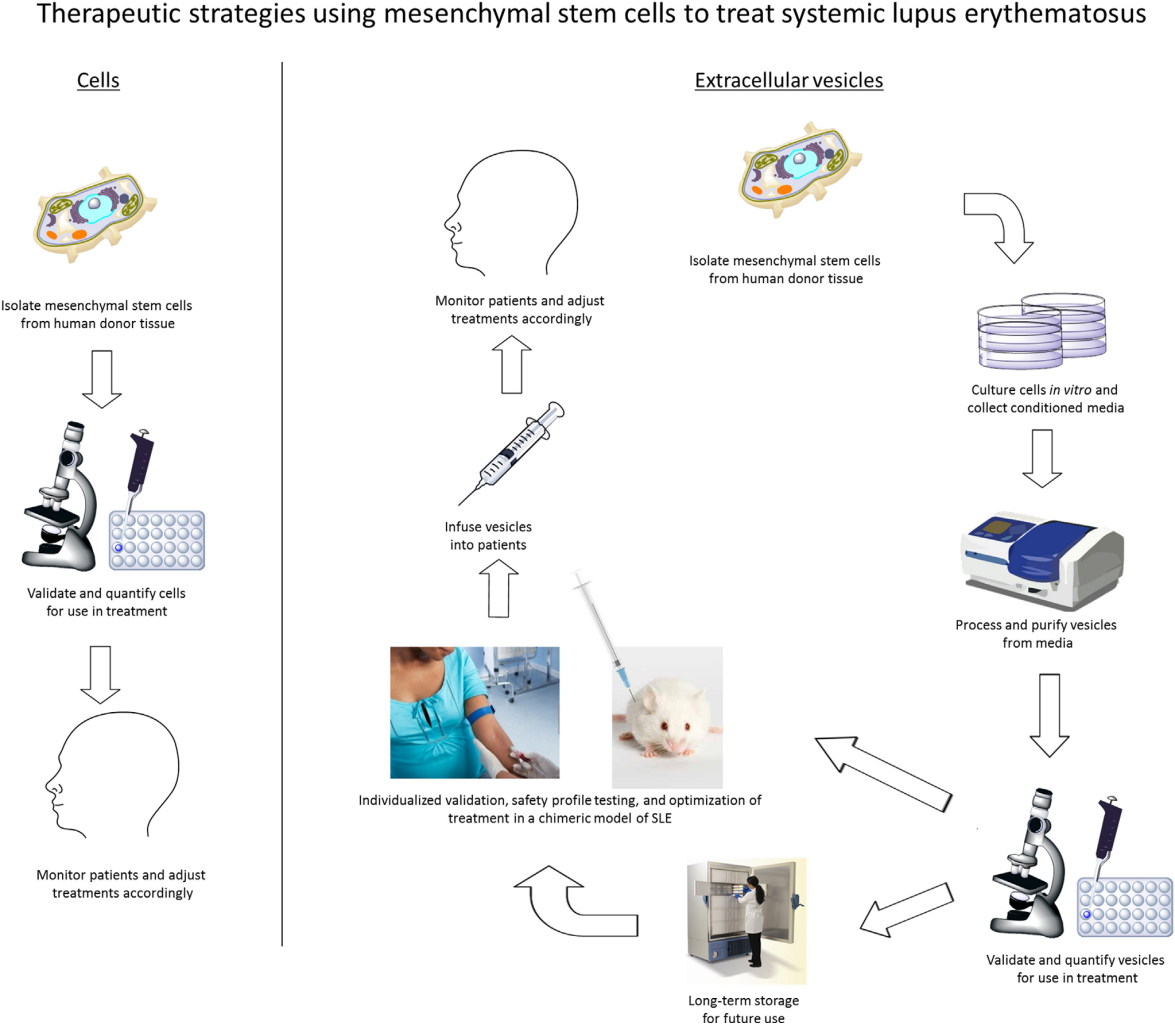
**Therapeutic strategies for the use of either mesenchymal stem cells (MSCs) or extracellular vesicles from MSCs to treat autoimmune diseases, including systemic lupus erythematosus**.

In summary, we propose that MSC-derived EVs are a feasible and more commercially viable cell-free therapeutic alternative to MSC therapy in the treatment of SLE. When compared to the conventional MSC therapeutic strategy commonly used today, the use of EVs to treat SLE will provide more flexibility as an off-the-shelf therapy, which will also permit individualized preclinical validation and safety-profile testing prior to patient infusion (Figure 1). In our future model of pharmacological application, MSCs would be isolated from human sources, confirmed by flow cytometry for expression of well-defined markers, and expanded *ex vivo*. EVs would be isolated from the conditioned media and screened by both proteomic and RNA profiling. These EV isolations can then be frozen until validation testing is complete. Additionally, the effects of MSC-derived EVs will be examined *in vivo* using a chimeric model of SLE developed recently in our laboratory (unpublished data) that is analogous to the human-mouse chimeric model we have previously developed for Sjögren’s syndrome (61). Following individualized chimeric model validation to determine treatment efficacy, MSC-derived EVs can be infused into SLE patients according to the data obtained in preclinical testing. Clinical responses would be monitored in patients and EV dosage, frequency, and duration will be adjusted individually. Our future work will develop and clinically evaluate this therapeutic model to treat SLE as well as analyze the mechanism of action used by MSC-derived EVs to suppress autoimmune-mediated inflammatory responses.

## Author Contributions

Wrote manuscript: JS and NY. Made figure: JH and NY. Edited manuscript; made substantial, direct and intellectual contribution to the work, and approved it for submission: JS, JH, GV, TW, HS, MY, RS, AB, SB, WJ, and NY.

## Conflict of Interest Statement

The authors declare that the research was conducted in the absence of any commercial or financial relationships that could be construed as a potential conflict of interest.
